# Diffuse Alveolar Hemorrhage Secondary to Ibrutinib Therapy in a Patient With Refractory Mantle Cell Lymphoma

**DOI:** 10.7759/cureus.8743

**Published:** 2020-06-21

**Authors:** Clifford B Locke, Frederick Lansigan

**Affiliations:** 1 Internal Medicine, Dartmouth-Hitchcock Medical Center, Lebanon, USA; 2 Hematology, Dartmouth-Hitchcock Norris Cotton Cancer Center, Lebanon, USA

**Keywords:** ibrutinib, diffuse alveolar hemorrhage, mantle cell lymphoma, bruton tyrosine kinase inhibitor, oncology, non-hodgkin lymphoma, adverse event, hematology, pulmonology

## Abstract

Ibrutinib is a Bruton tyrosine kinase inhibitor that is approved by the FDA for the treatment of mantle cell lymphoma and other hematological malignancies. Bruton tyrosine kinases promote platelet aggregation, and, therefore, bleeding is a common side effect of ibrutinib. At least half of patients taking ibrutinib experience a bleeding event, and up to 10% may experience major bleeding. Patient-specific factors that predict bleeding events remain unknown. This report describes a case of diffuse alveolar hemorrhage in a 67-year-old male taking ibrutinib for refractory mantle cell lymphoma. He was initially admitted to the hospital for recurrence of mantle cell lymphoma and evidence of tumor lysis syndrome, including acute kidney injury and hyperuricemia. He was taking aspirin prior to being hospitalized and was thrombocytopenic. A deep vein thrombosis was noted following admission, and the patient was started on enoxaparin. Two days after starting ibrutinib as an inpatient, the patient developed diffuse alveolar hemorrhage, which was ultimately fatal. Bronchoscopy with bronchoalveolar lavage ruled out infectious and other etiologies. To our knowledge, this is the first case of diffuse alveolar hemorrhage associated with ibrutinib. Based on the available literature, it is unclear if the patient’s recent aspirin use, concurrent enoxaparin, or thrombocytopenia was contributory. Further studies are necessary to clarify these patient-specific risks.

## Introduction

Ibrutinib is a Bruton tyrosine kinase (BTK) inhibitor that is approved by the FDA for the treatment of mantle cell lymphoma (MCL), chronic lymphocytic leukemia/small cell lymphoma (CLL/SLL), Waldenström’s macroglobulinemia, and chronic graft-versus-host disease [1,2]. BTK acts downstream of B-cell receptors to promote the differentiation, proliferation, and survival of B cells. Common adverse effects of ibrutinib include nausea, diarrhea, fatigue, and dyspnea. Other more serious side effects include infections, atrial fibrillation, and bleeding [3,4]. Real-word use of ibrutinib leads to discontinuation in 41% of patients, with intolerance being the leading cause in 50-60% of patients [5], 9% of which were due to bleeding complications.

Ibrutinib is known to increase bleeding risk by inhibiting BTK and Tec kinase in molecular pathways that regulate collagen-mediated platelet aggregation [6]. Based on results from prior studies, at least half of patients taking ibrutinib are expected to have a bleeding event [4,6]. Most of these bleeding events are grade 1 or 2 events based on the Common Terminology for Adverse Events criteria. Major bleeding events, defined as a grade 3 or higher, generally occur in less than 10% of patients and are rarely fatal. Long-term follow up of 111 patients taking ibrutinib for refractory MCL showed just seven major bleeding events [4]. Brown et al. [2] analyzed 15 studies consisting of 1,768 patients taking ibrutinib for MCL, CLL/SLL, or Waldenström’s macroglobulinemia. Of these patients, 88 (5%) suffered major bleeding events, and 27 of these cases were related to injury or trauma. The most common types of major bleeding otherwise were CNS events and gastrointestinal bleeding. Several other retrospective studies [5,7] suggested similar rates of major bleeding.

Thus, it is clear that ibrutinib increases the risk of bleeding events, though the risk of major bleeding remains controversial. Regarding pulmonary bleeding events, Brown et al. [2] identified two cases of hemoptysis, both of which were non-fatal [8,9]. Kreiniz et al. [10] reported pulmonary hemorrhage in the setting of severe opportunistic infection while taking ibrutinib. Here, we report a case of fatal diffuse alveolar hemorrhage (DAH) in a patient taking ibrutinib for MCL.

## Case presentation

A 67-year-old male with relapsed MCL presented to the outpatient department prior to starting therapy with ibrutinib 560 mg daily [3,11]. Previously, the patient had completed five/six cycles of the Nordic regimen [12], with repeat bone marrow biopsy after cycle 5 showing no active disease. He had been scheduled for high-dose chemotherapy with autologous stem cell rescue until biopsy of a right lower extremity mass about six weeks prior to his transplant showed relapse of disease. At his appointment, laboratory studies showed an LDH of 1,705 unit/L (normal: 110-220), uric acid of 11.2 mg/dL (normal: 3.5-8.5), and creatinine of 1.35 mg/dL (baseline: 1.0). Other notable labs included a hemoglobin of 9.9 g/dL (normal 13.7-16.5) and a platelet count of 36,000 per microliter, which were 13.4 g/dL and 217,000 per microliter, respectively, six weeks prior. Since the patient showed evidence of tumor lysis syndrome, even in the absence of systemic therapy, and cytopenias suggestive of disease progression, he was admitted to the hospital for treatment and to start ibrutinib as an inpatient.

The patient also had a medical history of coronary artery disease, peripheral artery disease, hypertension, hyperlipidemia, and hypothyroidism. Other medications included aspirin, metoprolol, morphine, gabapentin, trazodone, amlodipine, acyclovir, furosemide, levothyroxine, and rosuvastatin. His home aspirin was held following admission due to thrombocytopenia and anticipation of increased bleeding risk with ibrutinib therapy. Glucocorticoids and ibrutinib were started on hospital day 2 with the intention to add venetoclax once tolerating ibrutinib [13]. Concurrently, right greater than left lower extremity edema had been noted on examination, and a duplex ultrasound was consistent with deep vein thrombosis of the right popliteal vein. Low-dose enoxaparin 40 mg was started on hospital day 2 in lieu of full-dose anticoagulation due to the patient's thrombocytopenia [14].

**Figure 1:**
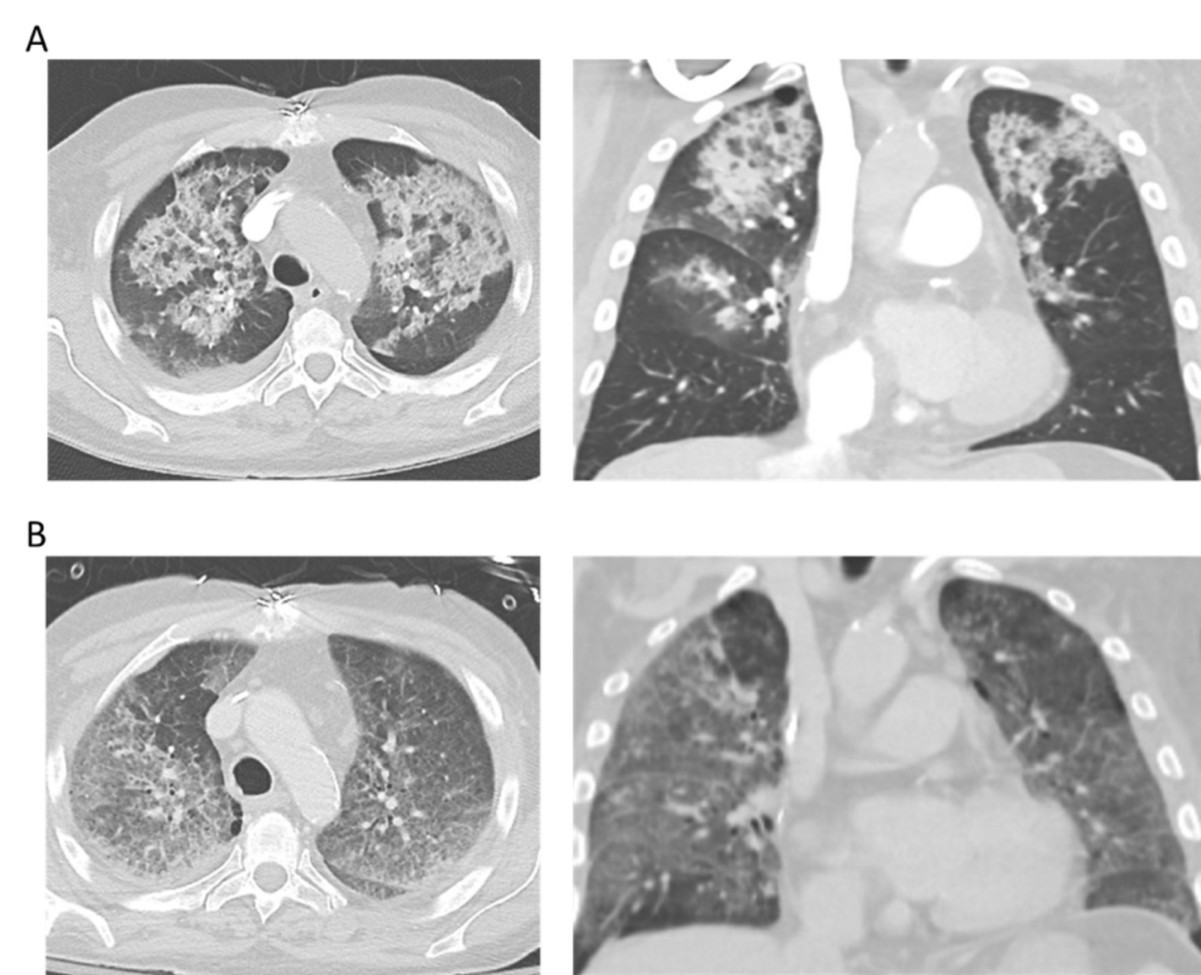
CT Angiogram of the Chest (A) Representative transverse (left) and coronal (right) images of the CT angiogram performed on hospital day 4 showing apical-predominant fluid-filled alveoli. (B) Representative images of the CT chest (with contrast) performed on hospital day 12 showing persistent ground-glass opacification of the lungs.

On hospital day 4, the patient reported dyspnea and coughed up a “quarter-sized” blood clot. At the time, his vital signs were stable, he was breathing room air comfortably, and examination was notable for faint crackles in the upper lung zones. Hemoglobin was 8.1 g/dL and platelet count was 26,000 per microliter. A CT angiogram of the chest was ordered (Figure 1) and showed small bilateral pleural effusions and fluid-filled alveoli in the upper lung zones. There was no evidence of pulmonary embolism. In the context of hemoptysis, fluid-filled alveoli were concerning for DAH. The following morning, the patient’s oxygen requirements rapidly increased and he was transferred to the ICU where he was sedated and intubated. He was transfused platelets, and both ibrutinib and enoxaparin were discontinued. Antibiotics were started in case of infection. Bronchoscopy with bronchoalveolar lavage (BAL) was performed. Visual inspection showed friable mucosa, blood, and secretions present in all segments. Serial lavages were performed and, as reported by the critical care physician, "each lavage was at least as bloody, if not more bloody, than the prior lavage, consistent with alveolar hemorrhage." Thus, the patient was diagnosed with acute respiratory failure secondary to DAH. Further workup revealed that blastoid cells were present in washings, but these were also present in the peripheral blood, and, therefore, their presence was not attributed to pulmonary involvement of MCL. BAL cultures showed normal respiratory flora, excluding acute pneumonia. Antibiotics were held and steroids were continued. He was extubated on hospital day 7 but was unable to be weaned from high-flow nasal cannula thereafter. Repeat CT scan on hospital day 12 showed persistent ground-glass opacification of the lungs (Figure 1). He expired two days later due to recurrent alveolar hemorrhage.

## Discussion

Our patient was a 67-year-old male with relapsed MCL who developed DAH within 48 hours of starting ibrutinib. To our knowledge, this is the first report of DAH associated with ibrutinib. We feel that ibrutinib was responsible for this event due to its known inhibition of platelet aggregation and, consequentially, increased risk of bleeding.

DAH is characterized by microvascular bleeding resulting from the accumulation of intra-alveolar red blood cells and hemosiderin-laden macrophages in BAL samples. The most common histology associated with DAH is pulmonary capillaritis [15]. Such microvascular damage is often associated with certain systemic diseases, including ANCA (anti-neutrophil cytoplasmic antibodies0 associated vasculitides, other forms of systemic vasculitis, and connective tissue disorders. Certain medications, including amiodarone and cytotoxic chemotherapeutic agents, have been implicated. DAH can also be precipitated by acute infection, coagulopathy, or malignancy [15]. Though hemosiderin-laden macrophages were not reported in our patient's cytology report, they may require up to 48-72 hours to accumulate [15], and his BAL was performed within 24 hours of symptom onset. Though there were small bilateral pleural effusions on CT scan, there was no report of interstitial markings. Additionally, a recent echocardiogram had shown a normal ejection fraction, and atrial fibrillation with rapid ventricular response was absent at the onset of hemoptysis; therefore, pulmonary edema was not felt to be causal to hemoptysis. DAH was the best diagnosis for the patient's clinical presentation, fluid-filled alveoli on CT scan, BAL findings, and rapid deterioration. Our patient had no known history of vasculitis, connective tissue disease, or other systemic illness that predisposed him to DAH. BAL cultures, as aforementioned, were inconsistent with acute pneumonia. Interestingly, blastoid cells were found in the BAL samples, which may suggest pulmonary involvement of MCL. However, since blastoid cells were also present on peripheral smear, this was not diagnostic. Though pulmonary involvement of MCL has been identified in a case report [16], it is extremely rare. In that case report, associated ground-glass opacities were seen on CT scan of the chest. Our patient had no such findings prior to the development of DAH. Our patient was also not receiving any medications that were associated with DAH and had not recently received cytotoxic chemotherapy. Without an alternative proximate cause, we attributed the patient's DAH to the initiation of ibrutinib.

The patient’s thrombocytopenia, recent aspirin use, and prophylactic anticoagulation likely contributed to the development of DAH. Predictors of major bleeding in patients taking ibrutinib have been studied but remain controversial. Studies have established an increased risk in patients taking vitamin K antagonists [6]. However, use of other anticoagulants or antiplatelet agents has not been definitively linked to increased risk of major bleeding with ibrutinib, and studies have shown conflicting data. An analysis of phase II and III trials of ibrutinib in CLL demonstrated that only 5 (2.9%) of 175 patients taking antiplatelet or anticoagulant agents concurrently with ibrutinib experienced major bleeding [17]. On the contrary, a small retrospective study [7] and long-term follow up of a phase II study of ibrutinib in refractory MCL [4], suggested that concurrent use of antiplatelet agents increases the risk of major bleeding with ibrutinib. In the study by Mock et al. [7], 70 patients who had taken ibrutinib were examined, and use of antiplatelet or anticoagulant medication conferred a 2.2-fold increased risk of major bleeding (hazard ratioL 2.2). Wang et al. [4] also reported an approximately two-fold increased risk (8% in those taking an antiplatelet and anticoagulant versus 4%). The aforementioned analysis by Brown et al. [2] also supported an increased risk of major bleeding while taking an antiplatelet agent with ibrutinib. However, the risk was no greater with ibrutinib than with other anticancer agents, suggesting that the effect was not specific to ibrutinib. In some of these same studies, thrombocytopenia was not identified as a risk factor for major bleeding with ibrutinib [2,7]. Real-world retrospective studies are elucidating more bleeding events than in clinical trials likely due to concurrent use of anticoagulants and anti-platelet agents, as well as more-comorbid conditions compared with a selected clinical trial population [5].

## Conclusions

Due to the low absolute risk of major bleeding with ibrutinib, identification of patients who are at high risk of major bleeding events, and guidelines for managing such patients, remain ambiguous. Many patients who had medical co-morbidities including thrombocytopenia and were on other anticoagulants or antiplatelet agents were excluded from initial studies. Further studies should be conducted to improve our ability to predict which patients are at the highest risk of major bleeding and to enhance our shared decision-making with patients.
